# Factors XI and XII in extracorporeal membrane oxygenation: longitudinal profile in children

**DOI:** 10.1016/j.rpth.2023.102252

**Published:** 2023-11-04

**Authors:** Joppe Drop, Natasha Letunica, Suelyn Van Den Helm, C. Heleen van Ommen, Enno Wildschut, Matthijs de Hoog, Joost van Rosmalen, Rebecca Barton, Hui Ping Yaw, Fiona Newall, Stephen B. Horton, Roberto Chiletti, Amy Johansen, Derek Best, Joanne McKittrick, Warwick Butt, Yves d’Udekem, Graeme MacLaren, Matthew D. Linden, Vera Ignjatovic, Chantal Attard, Paul Monagle

**Affiliations:** 1 Department of Paediatrics, Division of Paediatric Hematology, Erasmus Medical Centre—Sophia Children’s Hospital, Rotterdam, South Holland, The Netherlands; 2 Department of Paediatrics, Division of Paediatric Intensive Care and Paediatric Surgery, Erasmus Medical Centre – Sophia Children’s Hospital, Rotterdam, South Holland, The Netherlands; 3 Haematology Research, Murdoch Children’s Research Institute, Melbourne, Victoria, Australia; 4 Department of Paediatrics, The University of Melbourne, Melbourne, Victoria, Australia; 5 Department of Biostatistics, Erasmus University Medical Center, Rotterdam, South Holland, The Netherlands; 6 Department of Epidemiology, Erasmus University Medical Center, University Medical Center Rotterdam, Rotterdam, South Holland, The Netherlands; 7 Department of Clinical Haematology, The Royal Children’s Hospital, Melbourne, Victoria, Australia; 8 Department of Cardiac Surgery, The Royal Children’s Hospital, Melbourne, Victoria, Australia; 9 Department of Intensive Care, The Royal Children’s Hospital, Melbourne, Victoria, Australia; 10 Paediatric Intensive Care Research Group, Murdoch Children’s Research Institute, Melbourne, Victoria, Australia; 11 Department of Critical Care, The University of Melbourne, Melbourne, Victoria, Australia; 12 Department of Cardiac Surgery, Children’s National Heart Institute, Washington DC, USA; 13 Cardiothoracic Intensive Care Unit, National University Health System, Singapore; 14 School of Biomedical Sciences, The University of Western Australia, Perth, Western Australia, Australia; 15 Johns Hopkins All Children’s Institute for Clinical and Translational Research, St Petersburg, Florida, USA; 16 Department of Paediatrics, School of Medicine, Johns Hopkins University, Baltimore, Maryland, USA; 17 Kids Cancer Centre, Sydney Children’s Hospital, Randwick, New South Wales, Australia

**Keywords:** extracorporeal membrane oxygenation, factor XI, factor XII, infant, newborn

## Abstract

**Background:**

Extracorporeal membrane oxygenation (ECMO) is used in children with cardiopulmonary failure. While the majority of ECMO centers use unfractionated heparin, other anticoagulants, including factor XI and factor XII inhibitors are emerging, which may prove suitable for ECMO patients. However, before these anticoagulants can be applied in these patients, baseline data of FXI and FXII changes need to be acquired.

**Objectives:**

This study aimed to describe the longitudinal profile of FXI and FXII antigenic levels and function before, during, and after ECMO in children.

**Methods:**

This is a prospective observational study in neonatal and pediatric patients with ECMO (<18 years). All patients with venoarterial ECMO and with sufficient plasma volume collected before ECMO, on day 1 and day 3, and 24 hours postdecannulation were included. Antigenic levels and functional activity of FXI and FXII were determined in these samples. Longitudinal profiles of these values were created using a linear mixed model.

**Results:**

Sixteen patients were included in this study. Mean FXI and FXII antigenic levels (U/mL) changed from 7.9 and 53.2 before ECMO to 6.0 and 34.5 on day 3 and they recovered to 8.8 and 39.4, respectively, after stopping ECMO. Function (%) of FXI and FXII decreased from 59.1 and 59.0 to 49.0 and 50.7 on day 3 and recovered to 66.0 and 54.4, respectively.

**Conclusion:**

This study provides the first insights into changes of the contact pathway in children undergoing ECMO. FXI and FXII antigen and function change during ECMO. Results from this study can be used as starting point for future contact pathway anticoagulant studies in pediatric patients with ECMO.

## Essentials

• New contact activation inhibitors are promising in extracorporeal membrane oxygenation (ECMO).
• This is a prospective study of FXI and FXII antigenic and functional changes before, during, and after ECMO.
• FXI and FXII antigen and function drop after starting ECMO and recover after stopping ECMO.
• This study provides the first insights into contact pathway changes during ECMO in children.

## 1 Introduction

Extracorporeal membrane oxygenation (ECMO) is a form of modified cardiopulmonary bypass (CPB) and is used in children with refractory cardiac and/or pulmonary failure. Haemostatic complications including bleeding and thrombosis are among the most frequent complications in these patients [1]. Data from the Extracorporeal Life Support Organization Registry in 2020 demonstrated that hemorrhagic complications, including intracranial hemorrhage, were reported in up to 17.9% of neonatal and pediatric patients with ECMO [2]. Thrombotic complications arose in up to 35.5% of neonatal and 25.5% of pediatric patients [2]. The prevention of hemostatic complications is essential because these complications are associated with increased mortality and substantial morbidity [3,4].

Although ECMO has been used to support patients worldwide for over 50 years, the etiology of bleeding and thrombotic complications is not fully understood. Due to contact between blood and the surface of the ECMO circuit, the coagulation cascade is activated and hemostatic reserves are depleted, leading to a prothrombotic haemostatic state. To counteract this effect and to prevent thrombosis, anticoagulation is administered. The majority of ECMO centers use unfractionated heparin—and an increasing proportion bivalirudin—as the primary anticoagulant [5,6]. However, other anticoagulants targeting the contact activation pathway are emerging, including anti–factor XI (FXI) and anti–factor XII (FXII) [[7], [8], [9]].

The contact activation system consists of FXI, FXII, prekallikrein, and high-molecular-weight kininogen. The key glycoproteins FXI and FXII are appealing targets in ECMO because the contact pathway is strongly activated during ECMO support [10]. A previous study showed that targeting FXIIa was as effective as heparin in preventing circuit thrombosis in rabbits connected to pediatric ECMO circuits [11]. In addition, inhibition of the contact pathway is not associated with an increased risk of bleeding complications [7,12].

Before these promising new anticoagulants can be applied in clinical practice, it is important to understand how the concentration and function of these proteins change throughout the course of ECMO. Antigenic and functional FXI and FXII levels in children before, during, and after ECMO support are not known. Therefore, this study aims to describe the longitudinal profile of antigenic and functional FXI and FXII levels before, during, and after ECMO in children.

## 2 Methods

This is a prospective observational study in neonatal and pediatric patients with ECMO (<18 years) from the pediatric intensive care unit of the The Royal Children’s Hospital (RCH) between May 2017 and November 2020. Patients were included and data were collected according to a previously established protocol [13]. This study was approved by the RCH Ethics in Human Research Committee (HREC number: 35252). Written informed consent was obtained from legal guardians of all enrolled participants.

### 2.1 Patient selection and data and sample collection

All patients with venoarterial ECMO (VA-ECMO) and with sufficient plasma volume (>550 μL) available before ECMO initiation and on day 1, day 3, and after ECMO decannulation were included in this study. A total of 1.4 mL of blood was collected from the arterial lines into S-Monovette tubes containing sodium citrate and blood at a ratio of 1:9 (Sarstedt). Blood samples were centrifuged at 2500 *g* for 10 minutes at 20 °C (Biofuge Primo R) and stored at −80 °C until further processing. Frozen plasma samples were thawed at 37 °C in a water bath and were homogenized by gentle inversion. Samples were then tested within 2 hours of thawing. Reference ranges were derived from previous studies in healthy children [14,15]. The following clinical data were obtained: age, gender, ECMO indication, duration of ECMO support, bleeding and thrombotic complications, and survival to hospital discharge. Additionally, patients were classified (post-CPB vs non-CPB) based on whether they had been on CPB within 24 hours prior to initiation of ECMO. Neonatal patients were defined as all patients aged <30 days, infants were all patients between 31 days and 1 year of age, and children were defined as patients >1 year of age at ECMO initiation.

### 2.2 FXI and FXII concentration and function

Antigenic levels of FXI and FXII were determined using a matched-pair antibody set for human ELISA provided by Affinity Biologicals. Thawed plasma samples were added to Dade Hepzyme (Siemens Healthineers) after which the functional assay was performed on the STA-R Max analyzer (Diagnostica Stago) using STA—ImmunoDef FXI and FXII reagents (Diagnostica Stago). Cut-off values of antigenic and functional FXI and FXII deficiency were based on an earlier study in healthy children [14,15].

### 2.3 Statistical analysis

A linear mixed model was used to determine the longitudinal profile of FXI and FXII functional and antigenic assays. A random intercept was included in the linear mixed model to account for the within-subject correlations. The independent variables in the linear mixed model were timepoint (number of days since the start of ECMO, coded as a categorical variable) and age category (neonate, infant, and child). Repeated measures were taken into account by using the linear mixed model. The difference in FXI and FXII antigen and function between patients with and without CPB was calculated using the Mann–Whitney test. Mean values during ECMO were calculated using the mean of the values of days 1 and 3 during ECMO. A *P* value of.05 was considered the limit of significance in tests. All statistical analyses were performed using SPSS Statistics for Windows, version 28.0 (IBM).

## 3 Results

Sixteen out of the 96 treated patients were included in this study. Demographic, ECMO, and outcome parameters are depicted in Table 1.

**Table 1:** Demographic, ECMO, and outcome parameters.

Parameter	Patients with ECMO
Number of patients	16
Age, median (IQR; months)	2.5 (0.34-105.6)
Age groups, *n* (%)	
Neonate	7 (44)
Infant	4 (25)
Child	5 (31)
Male, *n* (%)	8 (50)
ECMO indication	
Cardiac, *n* (%)	13 (81)
Respiratory, *n* (%)	2 (13)
Other, *n* (%)	1 (6)
Duration of ECMO support, median (IQR; days)	7.2 (4-11.5)
Pre-ECMO cardiopulmonary bypass, *n* (%)	11 (69)
Patients with major bleeding complications, *n* (%)	8 (50)
Patients with thrombotic complications, *n* (%)	0 (0)
Survival to hospital discharge, *n* (%)	13 (81)

ECMO, extracorporeal membrane oxygenation; *n*, number.

Based on the linear mixed model, the mean antigenic FXI levels on day 1 (7.9 U/mL) and after ECMO decannulation (8.8 U/mL) were significantly higher than the average level on day 3 (6.0 U/mL; *P* = .041 and *P* = .037). The mean functional FXI levels on day 1 (47.9%) and day 3 (49.0 %) of ECMO support were significantly lower than the average functional FXI level after ECMO decannulation (66.0%; *P* = .002 and *P* = .004; Figure A). The mean antigenic FXII level before ECMO initiation (53.2 U/mL) was significantly higher than the average level on day 3 (34.5 U/mL; *P* = .002) and after ECMO decannulation (39.4 U/mL; *P* = .022). The average FXII functional assay was significantly higher before start of ECMO (59.0%) compared to day 1 of ECMO support (48.8%; *P* = .041; Figure B). The mean antigenic and functional activity and the number of patients with antigenic and functional FXI and FXII deficiency across timepoints are presented in Table 2. Median pre-ECMO antigenic FXI and FXII in children with (FXI: 6.1 U/mL; IQR: 4.2-9.3 U/mL; and FXII: 40.9; IQR: 22.5-78.6 U/mL) and without (FXI: 9.1 U/mL; IQR: 4.5-13.6 U/mL; and FXII: 63.8 U/mL; IQR: 34.4-96.5 U/mL) CPB were not significantly different (FXI: *P* = .036; FXII: *P* = .396). In addition, functional assays of FXI and FXII did not significantly differ between patients with (FXI: 52%; IQR: 39%-86%; and FXII: 55%; IQR: 38%-84%) and without (FXI: 54%; IQR: 51%-80%; *P* = .58; and FXII: 41%; IQR: 31-87.5; *P* = .58) CPB before ECMO initiation. Mean FXI and FXII antigenic and functional levels during ECMO were also not significantly different between patients with (FXI: 7.0 U/mL; FXII: 36.8 U/mL; FXI: 55.5%; FXII: 59.5%) and without (FXI: 7.2 U/mL; *P* = .44; FXII: 30.7 U/mL; *P* = .44; FXI: 45.3%; *P* = .23; FXII: 42.0%; *P* = .08) major bleeding complications. None of the patients suffered from a thrombotic complication.

**Figure:**
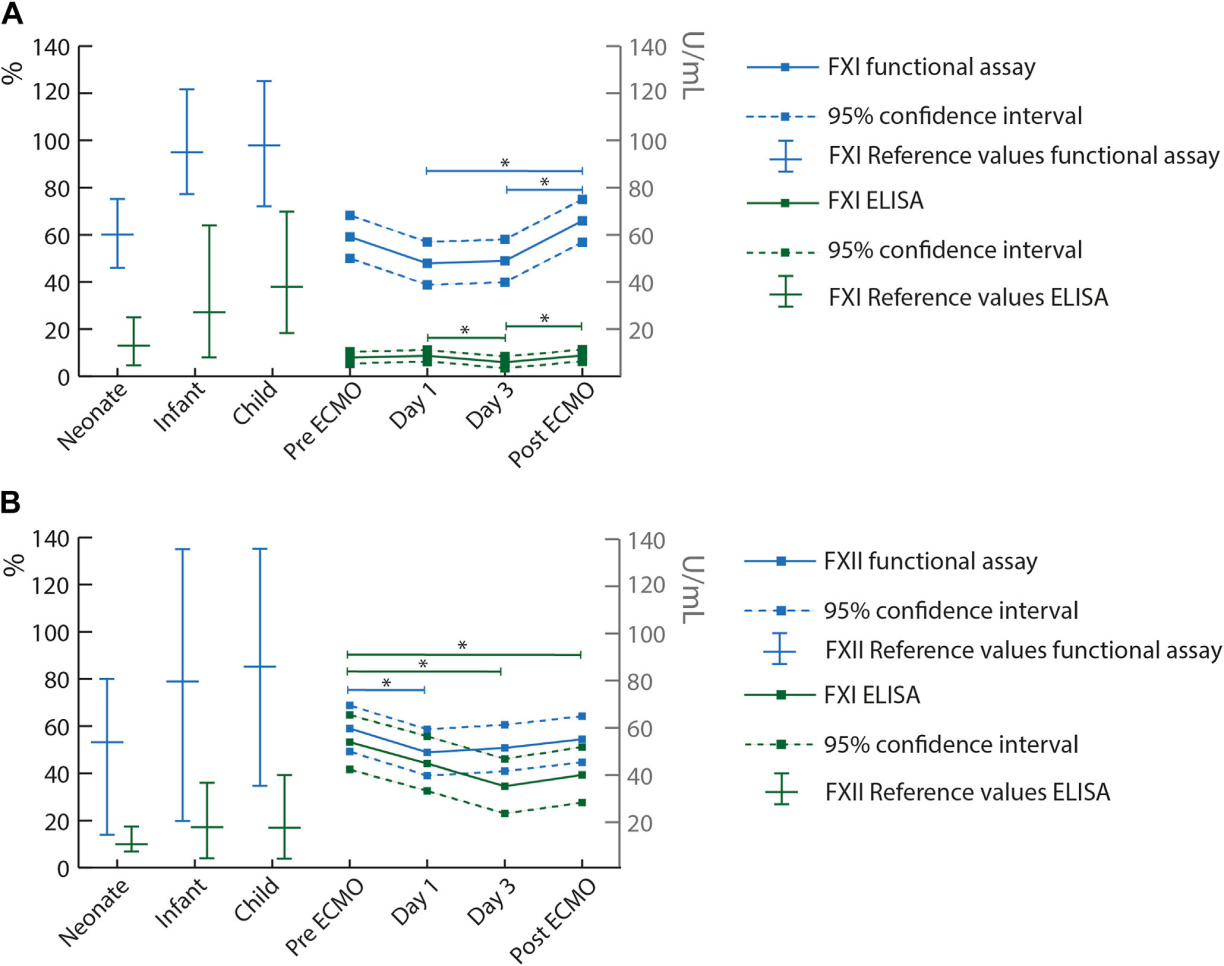
Median reference values with 95% CIs and longitudinal profiles of mean functional and antigenic FXI (A) and FXII (B) levels over time with corresponding 95% CIs and mean values in healthy children with 95% reference intervals. Data were based on the linear mixed model. ∗Significant difference (*P* < .05) between 2 timepoints. ECMO, extracorporeal membrane oxygenation; ELISA, enzyme-linked immunosorbent assay; FXI, factor XI; FXII, factor XII; mL, milliliter; U, units.

**Table 2:** Mean FXI and FXII levels with corresponding 95% CI over time. Data were based on the linear mixed model.

Variable	Pre-ECMO	Day 1	Day 3	Post-ECMO
FXI ELISA (U/mL)	7.9 (5.4-10.4)	8.9 (6.2-11.1)	6.0 (3.5-8.4)	8.8 (6.3-11.3)
*N* (%) in range	7 (44)	8 (50)	3 (19)	6 (38)
FXII ELISA (U/mL)	53.2 (41.6-64.7)	44.1 (32.6-55.7)	34.5 (23.0-46.1)	39.4 (27.6-51.1)
*N* (%) in range	16 (100)	16 (100)	16 (100)	16 (100)
FXI functional assay (%)	59.1 (50.0-68.2)	47.9 (38.8-57.0)	49.0 (39.9-58.0)	66.0 (56.9-75.0)
*N* (%) in range	12 (75)	8 (50)	8 (50)	14 (87)
FXII functional assay (%)	59.0 (49.2-68.8)	48.8 (39.0-58.6)	50.7 (40.9-60.5)	54.4 (44.6-64.2)
*N* (%) in range	15 (94)	16 (100)	15 (94)	15 (94)

ECMO, extracorporeal membrane oxygenation; ELISA, enzyme-linked immunosorbent assay; FXI, factor XI; FXII, factor XII; *N*, number; mL, milliliter; U, units.

## 4 Discussion

In this cohort, we demonstrated that pediatric ECMO patients have reduced antigenic levels of FXI compared to healthy children before, during, and after ECMO. In addition, half of patients had FXI functional deficiency and the longitudinal profile showed that mean functional FXI levels decreased after ECMO initiation, remained stable during ECMO, and significantly increased after ECMO cessation. None of the patients had antigenic FXII deficiency at any time point and functional FXII deficiency was rare. However, the longitudinal profile showed a significant decrease in antigenic and functional FXII levels after ECMO initiation.

The reduced antigenic levels of FXI after ECMO initiation might be explained by consumption of FXI as the contact pathway of the coagulation cascade is predominantly activated during ECMO [10]. This pattern also suggests that ECMO plays a role in the development of functional FXI deficiency. Interestingly, antigenic levels did not show the same pattern as functional FXI levels. With wider variation in FXI results, there might be a wider response to FXI inhibition. As a consequence, FXI inhibition may require patient-specific dosing strategies or at least an ability to monitor the overall antithrombotic effect of FXI inhibitors. Further studies are needed to investigate the longitudinal profile and deficiency of FXI to determine whether specific groups of patients (eg, cardiac surgical patients) have characteristic profiles of FXI over time. Antigenic or functional FXI and FXII levels have not been described in critically ill patients not on ECMO support.

In this study, antigenic and functional FXII deficiencies were rare. As a consequence, inhibition of FXII to achieve an anticoagulant effect might be more predictable. However, the longitudinal profile showed a significant decrease in antigenic and functional FXII levels after ECMO initiation. Activation of the contact pathway due to exposure of blood to the foreign surface of the ECMO circuit may lead to consumption of FXII. However, multiple factors can account for changes in clotting factors during ECMO, including heamodilution, renal replacement therapy, and underlying liver dysfunction [16]. After ECMO decannulation, antigenic and functional FXII levels recovered. This finding suggests that ECMO plays a role in antigenic and functional FXII level alterations. Of note, despite a significant decrease in FXII antigen and function during ECMO, antigenic or functional deficiency was rare in this cohort. It is not known whether the decreasing trend of FXII continues after the third day of ECMO support. This might have implications for FXIIa inhibition during prolonged ECMO support. Therefore, future studies need to elucidate the incidence and longitudinal profile of FXII deficiency after the third day of ECMO to evaluate the possible consequences for FXII inhibition during prolonged ECMO support.

Anticoagulation during ECMO support is one of the potential applications of the newly developed contact pathway inhibitors since this pathway is strongly activated during ECMO support [10]. The main benefit of contact pathway inhibitors is that they do not apparently increase the risk of bleeding while still exerting an anticoagulant effect. FXI and FXII inhibitors have been tested in *in vitro* studies [17,18]. However, the clinical use of direct target coagulation factor inhibition is contingent on understanding the antigenic and functional level of that target in health and disease [19]. The present study is the first study to provide insights into changes of the contact activation pathway of coagulation before, during, and after ECMO treatment in children. The strengths of this study are its prospective design and the fact that individual patients have been followed up over time. However, this study is limited by its small sample size, hampering subgroup (eg, different age groups), and multifactorial analyses. In addition, no additional plasma volume was available to determine FXI:SERPIN and FXII:SERPIN complexes, which could have been used to support the hypothesis of consumption of coagulation factors. Moreover, this cohort may be subject to selection bias, as there were no samples obtained from patients who did not survive. In addition, data about the ethnical distribution of the cohort were not available. As a consequence, information on sociocultural determinants of health could not be included. However, this study provides an important first insight into changes in the contact pathway in children undergoing ECMO support and forms the basis for future studies. Future studies can elucidate the correlation with clinical outcomes and the association with coagulation screening tests, and provide evidence for the suitability for treatment with contact pathway inhibitors in pediatric patients with ECMO.
